# Improvements in Timely Care and Patient-Reported Outcomes for Breast Cancer: A Seven-Year Southern Brazilian Cohort Analysis

**DOI:** 10.3390/healthcare14060786

**Published:** 2026-03-20

**Authors:** Rafaela Munari da Silva, Mauricio Santiago Soper, Larissa Conrado Martins, Isadora Ramos de Sousa, Juliana Haider Neves, Danica Reis Alcântara Soares, Carlos Emanuel Antunes Maciel, Gabriela Pacheco dos Santos, Andrey Roque de Oliveira, Isadora de Oliveira Severo Cardona, Samanta Brangel Pereira, Mariana Allende dos Santos, Arthur Pille, Juçara Gasparetto Maccari, Mohamed Parrini Mutlaq, Luiz Antônio Nasi, Jonas Michel Wolf

**Affiliations:** 1 Nursing Program, Faculdade de Ciências da Saúde Moinhos de Vento (FACSMV), Porto Alegre 90560-003, RS, Brazil; rafaela.munarisilva@hmv.org.br (R.M.d.S.); jonas.wolf@hmv.org.br (J.M.W.); 2 Hospital Moinhos de Vento (HMV), Porto Alegre 90035-000, RS, Brazil; samanta.pereira@hmv.org.br (S.B.P.); mariana.allende@hmv.org.br (M.A.d.S.); arthur.pille@hmv.org.br (A.P.); jucara.maccari@hmv.org.br (J.G.M.); mohamed.parrini@hmv.org.br (M.P.M.); luiz.nasi@hmv.org.br (L.A.N.)

**Keywords:** breast cancer, patient reported outcome measures, quality of life

## Abstract

**Highlights:**

**What are the main findings?**

- Patient-reported outcomes improved across all domains, including quality of life and body perception.
- A significant reduction was observed in the time from diagnosis to treatment initiation.

**What are the implications of the main findings?**

- Streamlined care pathways and early rehabilitation models can improve both treatment timeliness and patient-reported outcomes in breast cancer care.
- Expanding access to multidisciplinary, guideline-adherent oncology services may reduce disparities and enhance quality of care in middle-income settings.

**Abstract:**

**Background/Objective:** Breast cancer is one of the leading diseases affecting the Brazilian population and is often diagnosed at advanced stages. Due to its heterogeneity, treatment involves multiple therapeutic modalities, such as chemotherapy, hormone therapy, immunotherapy, and radiotherapy. The aim of this study was to characterize the profile of patients undergoing treatment for breast cancer in a private hospital in southern Brazil, and to assess the physical and psychological effects associated with different therapeutic modalities. **Methods:** An ambidirectional longitudinal cohort study was conducted from September 2018 to December 2024, incorporating retrospective data since 2013. Clinical and therapeutic data were collected, and Patient-Reported Outcome Measures (PROMs) using the QLQ-C30 Summary Score (QLQ-BR23, FACT-ES, BREAST-Q, LMC21) and Symptom Global Score questionnaires were analyzed using mixed-effects models to evaluate physical, emotional, cognitive, social, and overall quality-of-life domains, as well as body image. The temporal trend of time-to-treatment was assessed via linear regression. **Results:** Among 871 individuals evaluated, 98.4% were female, and invasive ductal carcinoma was the predominant histological type (75.1%). Radiotherapy was one of the most frequently used treatment modalities (39.2%), while immunotherapy had the lowest usage rate (2.7%). A significant reduction in the time between diagnosis and initiation of treatment was observed from 2013 to 2024 (from 21.0 to 10.9 days; *p* < 0.01), reflecting improvements in healthcare services. Mixed-effects models for PROMs indicated significant improvements across all assessed domains (*p* < 0.01) over the 48-month follow-up, despite a median follow-up of 22 months. **Conclusions:** High-quality and timely oncological care provided to breast cancer patients in a private hospital in southern Brazil demonstrates the implementation of a dynamic, agile, and human-centered care model, contributing to improved clinical and patient-reported outcomes validated by robust longitudinal analysis.

## 1. Introduction

Breast cancer is one of the most prevalent neoplasms worldwide [1], with the state of Rio Grande do Sul ranking third in Brazil in terms of incidence, reaching a rate of 62.7 cases per 100,000 women in 2024 [2]. It is also among the most heterogeneous cancers, capable of manifesting differently in each patient and requiring a range of therapeutic approaches [3]. These include surgery, chemotherapy, hormone therapy, radiotherapy, and immunotherapy [4,5,6,7,8]. Unfortunately, all these treatments share a common feature: negative side effects that can significantly impact the patient’s quality of life [8,9].

In Brazil, breast cancer is often diagnosed at advanced stages [10], which increases the need for more aggressive treatments, such as total or partial mastectomies and chemotherapy [8]. While effective in controlling advanced disease, these therapies are associated with intense side effects, as well as disfiguring and sometimes debilitating consequences [8,9,11]. Furthermore, while Federal Law No. 12.732/2012 (the “60-day law”) established a maximum timeframe for treatment initiation in the Brazilian Unified Health System (SUS), barriers to timely care persist in the public sector. In contrast, private healthcare institutions often implement streamlined care pathways that may offer superior agility in diagnosis and treatment initiation.

Among the available treatment options, immunotherapy stands out for its promising results in breast cancer management [7]; radiotherapy is a well-established approach in oncological control, sometimes capable of avoiding total mastectomies [5,8]; and hormone therapy, primarily indicated for hormone receptor-positive tumors, helps to reduce the proliferation of cancer cells [11].

In this context, it becomes essential to assess the impact of treatments from the patient’s perspective. For this purpose, standardized tools known as Patient-Reported Outcome Measures (PROMs), validated by the International Consortium for Health Outcomes Measurement (ICHOM), are used, which enable direct assessment of the patient’s perception of their health status, quality of life, and the physical and emotional impacts of the disease and its treatment. In the context of breast cancer, PROMs have proven to be essential for understanding how therapeutic interventions affect patients’ well-being. These tools support personalized care, encourage active listening, and contribute to a more humanized model of care focused on the individual experience with the disease. Furthermore, PROMs provide crucial insights for healthcare professionals to adjust and manage treatment more sensitively and effectively [12].

Therefore, this study aims to characterize the clinical and therapeutic profile of breast cancer patients treated at a private hospital in southern Brazil through an ambidirectional cohort study. The design integrates a prospective arm conducted between September 2018 and December 2024 with a retrospective analysis of data from patients treated since 2013 to evaluate the physical and psychological impacts associated with the different therapeutic modalities used. The investigation considered aspects such as clinical staging, molecular subtypes, therapeutic strategies, and clinical outcomes, with emphasis on overall survival and disease-free survival.

## 2. Materials and Methods

### 2.1. Study Design and Participant Selection

**Figure 1:**
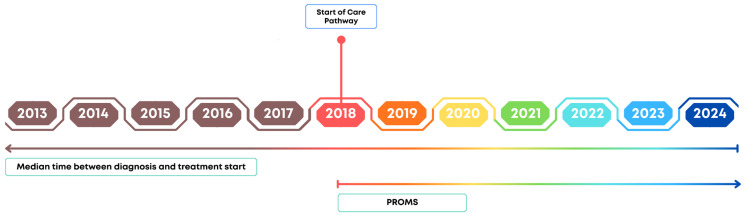
Timeline of the study phases and main outcomes assessed by year (2013 to 2024). From 2013 to 2017, only the median time between diagnosis and treatment initiation was collected; from 2018 onward, PROMs were also collected.

Post-discharge outcomes were assessed through an ambidirectional longitudinal cohort of 871 patients diagnosed with breast cancer, using standardized sets developed by ICHOM. This study integrated retrospective data (since 2013 to 2024) with a prospective cohort followed from September 2018 to December 2024 (Figure 1). This study was conducted at the Clinical Practice Management Office of Hospital Moinhos de Vento and approved by the institution’s Research Ethics Committee (CAAE: 66335222.6.0000.5330).

Participants included men and women over 18 years old diagnosed with invasive ductal carcinoma, ductal carcinoma in situ, and/or invasive lobular carcinoma. Enrollment occurred at the time of hospital admission. Patients were excluded if their main diagnosis were lobular carcinoma in situ, phyllodes tumor, other fibroepithelial lesions (including fibroadenoma), bilateral breast cancer, recurrent disease, or were undergoing adjuvant therapy at the time of study inclusion. Data collection included baseline clinical information and Patient-Reported Outcome Measures (PROMs) collected at T0 (baseline) and at follow-up intervals: 6, 12, 24, 36, and 48 months (T6, T12, T24, T36, and T48). The median follow-up time was 22 months. To assess potential attrition bias, response rates were monitored at each time point: T0 (100%), T6 (88%), T12 (82%), T24 (75%), T36 (70%), and T48 (65%).

### 2.2. Study Procedures and Data Collection

Data were collected through medical records and PROMs. The PROMs were assessed using the European Organization for Research and Treatment of Cancer (EORTC) QLQ-C30 Summary Score (QLQ-BR23, FACT-ES, BREAST-Q, LMC21) and the Symptom Global Score, each of them validated for the Brazilian population. Questionnaires were administered via email, telephone, and/or WhatsApp. All data were recorded in the REDCap platform. Any “alarming” responses triggered automated alerts for the attending physician, enabling active follow-up of clinical demands.

### 2.3. Sampling

A non-probabilistic convenience sampling method was used, including all eligible patients (*n* = 871) treated during the study period.

### 2.4. Statistical Analysis

Data were analyzed using SPSS^®^ version 25.0 and R software version 4.5.2. The distribution of quantitative variables was assessed using the Kolmogorov–Smirnov test. Medians and interquartile ranges (IQR) were calculated, and Mann–Whitney U or Kruskal–Wallis tests were applied for group comparisons (e.g., comparing clinical stages and surgical types).

To account for the longitudinal nature of the PRO data and handle missing values, mixed-effects models (mixed-effects linear regression) were employed. These models evaluated changes in PROM domains over time, adjusting for potential confounders such as age and baseline clinical stage. Additionally, to analyze the temporal trend of the interval between diagnosis and treatment initiation (2013–2024), a linear regression analysis was performed, using the year of diagnosis as a continuous predictor to confirm the statistical significance of the observed reduction. All estimates were two-tailed, with the significance level set at α = 5% (*p* < 0.05).

## 3. Results

This study analyzed a total of 871 patients (Figure S1), the vast majority of whom were female (98.4%). White skin color was predominant, representing 96.4% (840/871) of the sample. Regarding marital status, 61.5% (536/871) of the patients were married. The median age was 57 years (IQR: 25.0–96.0). The median time since the start of treatment was 656.2 days (IQR: 7.0–1442.0), and the interval between diagnosis and the beginning of treatment had a median of 27.0 days (IQR: 0.0–323.0). Concerning tumor histology, invasive ductal carcinoma was the most prevalent type (75.1%), followed by ductal carcinoma in situ (29.1%) and invasive lobular carcinoma (14.5%). The sum of histological percentages exceeds 100% because several patients presented mixed histological components (e.g., IDC with an in situ component) (Table 1).

Table 1 presents the types of treatments administered. Regarding surgical treatment, 88.4% of patients (756/855) underwent some form of surgery, and 95.2% of those (720/756) also underwent an axillary procedure. Immediate breast reconstruction was performed in 88.6% (670/756), at least 76.9% of the total sample, a rate significantly higher than the Brazilian national average, reflecting the institutional practice of this private center, possibly higher due to missing data. Delayed reconstruction was conducted in 4.2% of patients. Radiotherapy was administered to 39.2% of the patients (332/846), 99.1% of those in the adjuvant setting (329/332).

Chemotherapy was used in 41.5% of patients (351/846); of those, 45.8% received it as adjuvant therapy and 55.3% as neoadjuvant. Among chemotherapy agents, taxanes were used by 83.1% (285/351), followed by anthracyclines with 59.9% (208/351). Hormonal therapy was prescribed to 39.0% of patients (330/846), with aromatase inhibitors being the most prescribed among them (60.6%). Targeted therapy was administered to 13.1% of patients (111/846), with HER-2-directed treatment being the most common among them (87.9%). Immunotherapy was used in 2.6%; this low prevalence suggests either an unexpectedly small proportion of triple-negative breast cancer patients within the sample or, less likely (due to the patient profile of the private hospital), the existence of barriers to treatment access.

Among patients who underwent reoperations (*n* = 203), 73.4% were breast reconstructions (149/203). Regarding complications within 90 days post-treatment (*n* = 142), 28.2% required surgical, radiological, or endoscopic intervention. Complications were classified following the ICHOM recommendations. The 40.1% originally classified as “another type” consisted primarily of wound seromas (15.5%), minor surgical site infections requiring oral antibiotics (12.7%), and localized flap necrosis (11.9%). Severe complications leading to ICU admission were recorded in 2.8% of cases, while 5.6% of patients experienced complications that led to complete discontinuation of treatment. Death due to treatment-related complications occurred in 0.7% of cases (Table 2).

**Figure 2:**
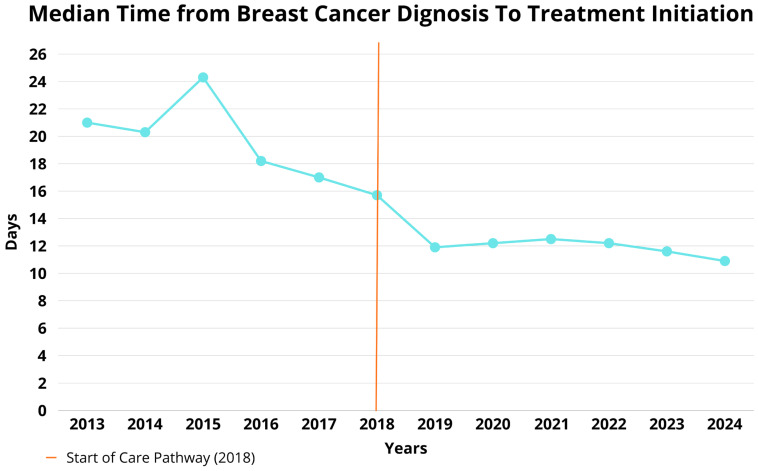
Temporal evolution of the time between diagnosis and start of treatment (linear regression, *p* < 0.01).

Figure 2 illustrates the temporal trend of the median time between breast cancer diagnosis and the start of treatment. To confirm this trend, a linear regression analysis was performed using the year of diagnosis as a continuous predictor, which revealed a significant annual reduction (from 21.0 days in 2013 to 10.9 days in 2024; a difference of 51.9%, beta = −1.02, *p* < 0.01). This reduction is probably due to substantial improvements in institutional agility and the performance of the care service, facilitating more dynamic treatment initiation.

**Figure 3:**
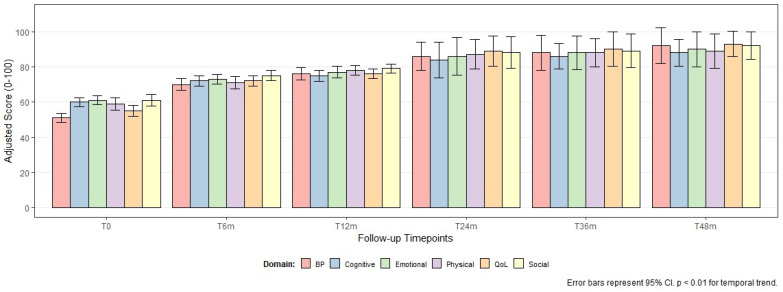
Longitudinal evolution of Patient-Reported Outcome Measures (PROMs) over 48 months of follow-up at different follow-up times (T0: baseline, T6m: 6 months, T12m: 12 months, T24m: 24 months, T36m: 36 months, T48m: 48 months). The values represent adjusted mean scores derived from linear mixed-effects models, accounting for repeated measures and adjusting for age and clinical stage. Error bars represent 95% confidence intervals (95% CI), with increased variance observed from 24 months onwards, reflecting the statistical impact of attrition bias in long-term follow-up. Significant improvements (*p* < 0.01) are observed across all domains: Physical, Emotional, Cognitive, Social, overall Quality of Life (QoL), and Body Perception (BP). QoL: Quality of Life; BP: Body Perception.

Along these lines, the analysis of PROMs over a 48-month follow-up period (Figure 3) demonstrated significant improvements across all evaluated domains (physical, emotional, cognitive, social, overall quality of life, and body image perception). To account for the longitudinal nature of the data and handle missing follow-up values (attrition), linear mixed-effects models were applied. The models confirmed that these improvements were statistically significant over time (*p* < 0.01 for all domains), even after adjusting for baseline clinical stage and patient age. The data presented in Figure 3 represent mean scores with 95% confidence intervals, reflecting a consistent recovery in patient-reported well-being throughout the care pathway.

## 4. Discussion

The data obtained in this study allow for a comprehensive analysis of the clinical-demographic, therapeutic, and outcome profiles of breast cancer patients treated at a private hospital in southern Brazil. The predominance of women with a median age of 57 years is consistent with national epidemiological studies, which report a higher incidence between the ages of 50 and 69, with invasive ductal carcinoma being the most common histological subtype [13]. European and North American studies also confirm this age trend, although variations occur depending on socioeconomic factors and access to healthcare [14].

The high rate of surgical procedures (88.4%), particularly immediate breast reconstruction (78.4%), reflects a current oncological practice trend focused on early rehabilitation and patient aesthetics and self-esteem. This finding aligns with other studies [15], which highlight that this approach, more frequent in private hospitals, still faces limitations in the Brazilian public health system (SUS) due to resource constraints and lack of specialized teams.

Regarding chemotherapy, the predominance of taxanes and anthracyclines aligns with national Clinical Protocols and Therapeutic Guidelines for Oncology [16], especially for locally advanced or high-risk tumors. Neoadjuvant administration, as seen in 54.7% of cases, is especially relevant for tumor downstaging and enabling breast-conserving surgeries [17]. It is estimated that approximately 40–60% of patients receive chemotherapy during treatment, depending on clinical staging and tumor molecular profile.

Despite the low frequency of immunotherapy (2.7%) and targeted therapy (13.1%) in this cohort, there is a growing trend toward the inclusion of these modalities in the Brazilian oncology landscape. For example, HER-2-directed therapy was used in only 13.1% of patients—a figure lower than in countries with broader access to molecular testing and biologics. In the United States, anti-HER2 therapies are standard in HER2-positive cases [6]. Similarly, the low use of immunotherapy reflects the global pattern, as indications remain limited to specific cases such as triple-negative tumors expressing PD-L1 [6]. Recent studies show that adding agents like atezolizumab in combination with nab-paclitaxel significantly improves progression-free survival in patients with metastatic disease [18]. Furthermore, hormonal therapy was used in approximately 40% of patients, mostly with aromatase inhibitors, reflecting the prevalence of luminal tumors. This aligns with the literature [19], which points to substantial benefits from adjuvant hormonal therapy in hormone receptor-positive patients.

Temporal analyses showed significant progress in reducing the time between diagnosis and the start of treatment (*p* < 0.01), reflecting improvements in care pathways and organizational advances following the implementation of a care line. This reduction may have a direct impact on clinical outcomes, as delays over 60 days are associated with lower overall survival [20]. Additionally, treatment delays are frequently reported as a major barrier in Brazil’s public healthcare system [10], highlighting the need for institutional policies aimed at faster diagnosis and treatment.

Reported complications (16.3%) and serious adverse events such as ICU admissions or treatment interruptions underscore the challenges of managing toxicities, especially in combined therapies. Therefore, close monitoring and multidisciplinary support are essential to minimize these events and ensure treatment adherence [9]. In this context, PROMs data also showed significant improvements in patients’ quality of life over time, especially in emotional, physical, and social domains, reinforcing the importance of integrated support and rehabilitation strategies during and after treatment and showing that psychosocial interventions play a crucial role in the overall recovery of cancer patients [9].

This study presents limitations that must be considered when interpreting the results. First, the sample may not be fully representative of other Brazilian populations, as data were collected from a single private hospital in southern Brazil, potentially reflecting a specific socioeconomic profile with access to a high standard of oncologic care. Furthermore, the lack of randomization among therapeutic groups may introduce bias, as clinical, anatomical, and individual preference factors may have influenced treatment decisions. Another important limitation is the lack of more detailed data on psychosocial variables and pre-existing comorbidities, which could directly impact treatment adherence and quality of life outcomes. Although the four-year longitudinal follow-up represents an improvement over cross-sectional studies, this period may still be insufficient to fully capture long-term effects of certain interventions, especially regarding body image, sexuality, and social reintegration. Finally, we encountered difficulty in consistently confirming the direct association between deaths and breast cancer, which compromises the accuracy of cause-specific mortality classification and reduces the reliability of cancer-specific survival analyses. Furthermore, the total number of events was very low (*n* = 10 deaths) throughout the follow-up period. Such a limited number of events makes a robust survival analysis impossible, including stable Kaplan–Meier estimates or Cox proportional hazards modeling. For these reasons, we considered it methodologically more appropriate not to report survival analyses in this study, even though they were originally included among the study objectives.

Considering the findings and existing gaps, future studies should deepen the analysis of long-term outcomes and explore strategies to expand access to innovative therapies, supporting the development of an increasingly efficient, equitable, and personalized oncology care model.

## 5. Conclusions

The significant improvement in PROMs over four years highlights the value of an integrated oncologic care approach with early rehabilitation, pointing to a transforming healthcare landscape in which quality of care and the patient experience take center stage. Challenges persist regarding treatment-related toxicities and limited access to targeted therapies and immunotherapy, underscoring the need to expand advanced diagnostic and therapeutic resources in Brazil. Thus, this study contributes not only to understanding the national oncology landscape but also to strengthening institutional strategies aimed at clinical excellence, equitable access, and a holistic appreciation of the journey of women with breast cancer.

## Figures and Tables

**Table 1:** Demographic, clinical and type of treatment in a sample of patients treated at a private hospital in southern Brazil.

Variables	Median (IQR *)	*n*	%
Female	-	857	98.4
White Skin Color	-	840	96.4
Married	-	536	61.5
Median Age (IQR)	57 (25.0–96.0)	-	-
Time relative to treatment (*n* = 871)			
Time since start of treatment	656.2 (7.0–1442.0)	-	-
Time between diagnosis and treatment	27.0 (0.0–323.0)	-	-
Type of tumor (*n* = 855)	-		
Ductal carcinoma in situ	-	249	29.1
Invasive ductal carcinoma	-	642	75.1
Invasive lobular carcinoma	-	124	14.5
Surgery (*n* = 855)		756	88.4
Axillary surgery	-	720	84.2
Delayed breast reconstruction	-	32	3.7
Immediate breast reconstruction	-	670	78.4
Radiotherapy (*n* = 846)	-	332	39.2
Adjuvant	-	329	99.1
Neoadjuvant	-	3	0.9
Chemotherapy (*n* = 846)	-	347	41.0
Adjuvant	-	159	45.3
Neoadjuvant	-	192	54.7
Chemotherapy type (*n* = 846)		-	-
Anthracycline	-	208	59.7
Taxanes	-	285	81.2
Platinum analogues/platinum-based	-	49	14.0
Other	-	38	10.8
Unknown	-	7	2.0
Hormone therapy (*n* = 846)		330	39.0
Adjuvant	-	303	91.8
Neoadjuvant	-	25	7.6
For stage IV patients	-	2	0.6
Hormone therapy type (*n* = 846)		-	-
Aromatase inhibitor	-	200	60.6
SERMs **	-	118	35.7
LHRH *** agonist	-	58	17.8
Other	-	3	0.9
Unknown	-	10	3.0
Targeted therapy (*n* = 846)	-	111	13.1
HER-2 ****	-	102	91.9
Immunotherapy	-	3	2.7
Cyclin inhibitor	-	6	5.4
Other	-	5	4.5

* IQR: interquartile range; ** SERMs: selective estrogen receptor modulators; *** LHRH: luteinizing hormone-releasing hormone; **** HER-2: human epidermal growth factor receptor 2.

**Table 2:** Profile of reoperations and complications within 90 days after treatment starts.

Variables	*n*	%
Reoperation (*n* = 203)		
Breast reconstruction surgery	149	73.4
Therapeutic mastectomy/adenomastectomy	21	10.3
Preventive mastectomy/adenomastectomy	14	6.9
Axillary dissection	11	5.4
Sectorectomy	2	1
Quadrantectomy	6	3
Complication within 90 days of treatment (*n* = 142)		
Complication requiring intervention (surgical, radiological, endoscopic)	40	28.2
Complication leading to prolonged hospitalization (>14 days)	8	5.6
Complication leading to unplanned readmission	7	4.9
Complication leading to ICU * admission	4	2.8
Complication leading to interruption of treatment	8	5.6
Complication leading to dose reduction	17	12.0
Complication leading to death	1	0.7
Another type of complication	57	40.1

* ICU: Intensive Care Unit.

## Data Availability

Data is unavailable due to privacy and ethical restrictions.

## Supplementary Materials

The following supporting information can be downloaded at: https://www.mdpi.com/article/10.3390/healthcare14060786/s1, Figure S1: Study Participants Flowchart.

## Abbreviations

The following abbreviations are used in this manuscript:

IQR	Interquartile Range
SERMs	Selective Estrogen Receptor Modulators
LHRH	Luteinizing Hormone-Releasing Hormone
HER-2	Human Epidermal Growth Factor Receptor 2
SUS	Sistema Único de Saúde
PROMs	Patient-Reported Outcome Measures
ICHOM	International Consortium for Health Outcomes Measurement
