# Supplementary figures and images for: Improvements in Timely Care and Patient-Reported Outcomes for Breast Cancer: A Seven-Year Southern Brazilian Cohort Analysis
Source: Healthcare (Basel). 2026 Mar 20;14(6):786. doi: 10.3390/healthcare14060786 (PMC13026368; doi:10.3390/healthcare14060786)

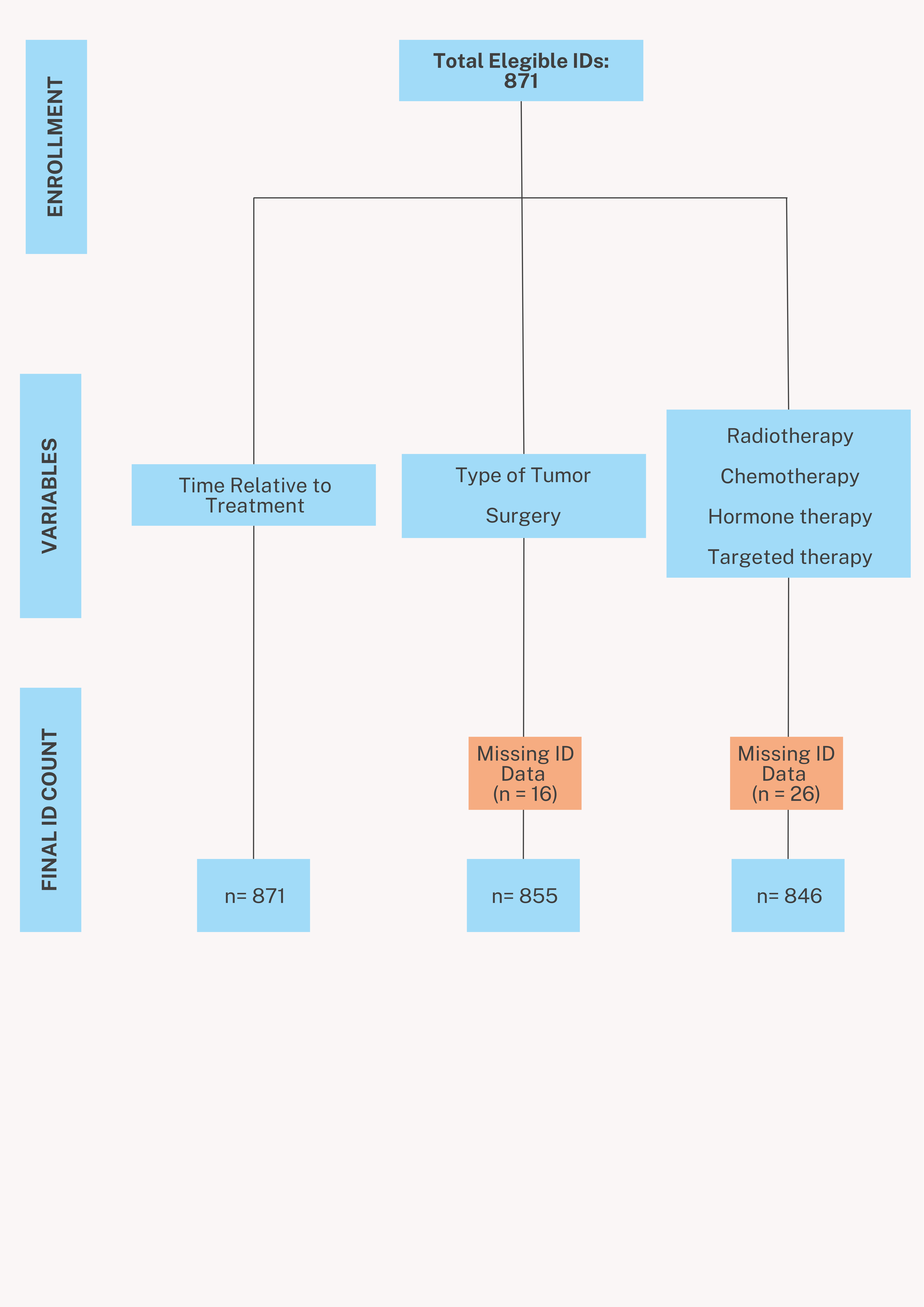

Supplement: Supplementary file 1 [file healthcare-14-00786-s001.zip › Figure S1.png]
